# Effects of inhaled hypertonic (7%) saline on lung function test in preschool children with cystic fibrosis: results of a crossover, randomized clinical trial

**DOI:** 10.1186/s13052-017-0376-6

**Published:** 2017-07-15

**Authors:** Raffaella Nenna, Fabio Midulla, Caterina Lambiase, Giovanna De Castro, Anna Maria Zicari, Luciana Indinnimeo, Giuseppe Cimino, Patrizia Troiani, Serena Quattrucci, Giancarlo Tancredi

**Affiliations:** 1 Department of Pediatrics and Infantile Neuropsychiatry, “Sapienza” University of Rome, Rome, Italy; 2 Cystic Fibrosis Centre-Department of Pediatrics and Infantile Neuropsychiatry, “Sapienza” University of Rome, Rome, Italy

**Keywords:** Cystic fibrosis, Hypertonic saline, Children, Therapy, Inhalation

## Abstract

**Background:**

This crossover, randomized, double-blind study (conducted over a 32-week period) was performed to determine, in clinically stable Cystic fibrosis (CF) preschool children: the effects of 7% inhaled hypertonic saline on spirometry and interrupter resistance technique (Rint), and the possible side effects.

**Methods:**

Twelve CF children (6M, mean age ± SD: 5.7 ± 0.8 yrs) were enrolled and randomly assigned to receive hypertonic saline (HS-4 ml 7% sodium chloride), or normal saline (NS-0.9% sodium chloride) twice a day. After a 16 weeks period, therapy was exchanged to allow all the patients enrolled in the study to carry out both treatments. Monitoring visits, spirometry (COSMED Quark PFT4 ergo) and Rint were scheduled at 0,4,16,20,32 weeks. At T0, spirometric measurements and Rint were performed immediately before and 30 min after the inhalation therapy. Salbutamol (400 mcg) was administered before the drug at each visit.

**Results:**

After a 16-weeks treatment with HS an improvement of FVC (*p* = 0.02) and a favorable trend of FEV1 were registered. A worsening of FEV_1_ (*p* < 0.0001) and of FEF25-75 (*p* = 0.019) were found in NS group. No differences were found in expiratory and inspiratory Rint in both groups. No serious adverse events occurred.

**Conclusions:**

Seven percent hypertonic saline therapy proved to be a useful and safe treatment in young CF children with clinically stable conditions.

**Trial registration:**

ISRCTN12345678.

## Background

Cystic fibrosis (CF) is the most common hereditary disease in Caucasians. It is caused by mutations in the CF transmembrane conductance regulator (CFTR) chloride channel. This mutations lead to an abnormal ion transport across the respiratory epithelium with a defective chloride secretion and excessive sodium absorption that finally cause dehydration of the airway surface liquid. The disruption of mucociliary clearance, apparently closely dependent on the presence of an inadequate volume of airway surface liquid, plays a basic role in the vicious cycle of airway obstruction, infection, inflammation and lung damage [1]. According to this hypothesis, inhaled hypertonic saline (HS) represents a well-documented therapy in adults [1, 2], as the already established but very expensive treatment with rhDNase [2, 3].

The effects of the HS on pulmonary function tests have been assessed in young children with CF [4–6], providing to be associated with an improvement in lung function and marked benefits with respect to exacerbations [7].

This crossover, randomized double-blind study was designed to determine as primary outcomes, in preschool children with CF: the effects of an inhaled hypertonic solution on spirometry (measured as follows: forced volume vital capacity – FVC, forced expiratory volume in 1 s - FEV1 and forced expiratory flow 25–75% - FEF25-75) and airways resistance assessed by The Interrupter technique (Rint). As secondary outcome the possible side effects of the treatment were evaluated.

## Methods

### Study population

Over the period September 2012 - September 2013, children with a confirmed diagnosis of cystic fibrosis by sweat test and genetic analysis, were enrolled.

Eligible subject included clinically stable preschool children with CF undergoing a simple therapy based on bronchodilator (salbutamol) and physiotherapy. Children were considered stable whether: FEV1, measured at screening, was at least 70% of predicted value for height, weight, age and sex, as well as the expiratory resistance was lower than 150% of predicted values [8]. Moreover, they should not be colonized with Burkholderia Cepacia, or have respiratory infections during the treatment or 2 weeks before and therapy with rhDNase or routinely use of antibiotics.

### Study design

**Fig. 1:**
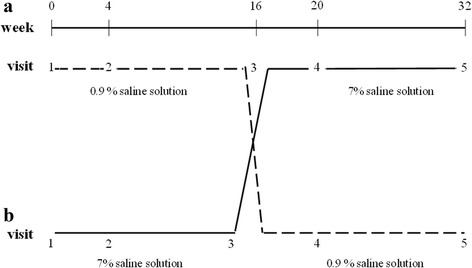
Study design: weeks and visits in the two groups of children

A crossover, double-blind randomized study was conducted over a 32-week period (Fig. 1). Randomization was performed using a randomization table. Patients were enrolled and assigned blindly to one of the group by the attending physician. Children were randomly assigned to receive a 4 ml treatment with hypertonic saline (HS – 7% sodium chloride), or normal saline (NS - 0.9% sodium chloride) twice daily. A bronchodilator (400 mcg of salbutamol) was administered 20 min before each inhalation of the study solution. After a 16 weeks period, therapy was exchanged to allow all the patients enrolled in the study to carry out both treatments.

Monitoring visits were scheduled at 0, 4, 16, 20, 32 weeks. At each visit, participants underwent history and physical examination, sputum collection with its microbiological examination and susceptibility testing, baseline spirometry and measurement of airway resistance by Rint.

Pulmonary function testing was performed with the COSMED Rocc Quark PFT4 ergo (Rome,Italy) device at the Cystic Fibrosis Centre, of the Pediatric Department, “Sapienza” University of Rome.

### Lung function test

Airway resistance was measured using the interrupter resistance technique (Rint). Each single interruption was considered acceptable when the trace of mouth pressure versus time showed the correct shape [9]. The Rint measurement was taken with the child sitting comfortably and voluntarily, during quiet spontaneous respiration, using a disposable mouthpiece with an antibacterial filter device. The child was instructed to put the nasal clip in place, closing the lips around the mouthpiece and placing the tongue under the mouthpiece. The researcher supported the face and chin of the child in order to avoid energy loss and reduce the effect of upper airway compliance. The head was held in neutral position. Researchers trained in the method performed the measurements [10].

At each visit, a bronchodilator (400 mcg of salbutamol) was administered via a metered dose inhaler and large volume spacer (Volumatic, Glaxo, UK) 20 min before the inhaled HS or NS administered using a pediatric aerosol nebulizer (OS80/P) aerosol therapy mask. Rint was performed before spirometry, as the deep inspiration preceding the forced expiratory maneuver required to perform spirometry could influence the bronchial tone. Spirometric measurements and Rint were performed immediately before the bronchodilator at the start of study. Spirometry measures were reported as absolute values and % predicted values, according to the global lung initiative reference data [11]. Rint measurements were taken according to the ATS/ERS recommendation [8]. Another spirometry and Rint were performed 30 min after the inhalation therapy (HS or NS) at T0, T4 and T16. Values obtained after the administration of the bronchodilator at T0 were used for starting values.

### Statistical analysis

All statistical calculations were performed using SPSS for Windows version 21.0 (SPSS, Inc., Chicago, Illinois, USA). A paired sample t-test was used to evaluate difference between mean values for each treatment at time 0 (T0), at 4 (T4) and 16 weeks (T16). Results were expressed as mean ± SD. Significance levels were set at ≤0.05.

All parents of children gave written informed consent and the protocol (prot. HS-2009) was approved by the Scientific Ethics Committee, Policlinico Umberto I.

## Results

Insofar as 1 child had respiratory infections, he was excluded. A total of 12 children (six males, mean age ± SD: 5.7 ± 0.8 years) with a confirmed diagnosis of cystic fibrosis were enrolled (Table 1).

**Table 1:** Anthropometric characteristics of the participants

Patients	
N°	12
Age (years)	5.7 ± 0.8 (range: 4–5.8)
Sex (Males/Females)	5/7
Height (cm)	116 ± 5.7
Weight (Kg)	21.4 ± 3.7
BMI (Kg/m^2^)	15.9 ± 1.7

All the 12 patients completed the entire protocol. At T0 no differences were found in terms of mean FVC, FEV1 and FEF25-75 as well as mean expiratory and inspiratory Rint between HS and NS groups. The sputum following administration of HS were almost unchanged during the study protocol (not colonization with Burkholderia Cepacia nor new respiratory infections). We did not record any exacerbations of respiratory infections in both groups.

At T0 after bronchodilator, significant improvements of FVC, FEV1 and FEF25-75 were found in both groups (HS: FVC: 1.36 ± 0.4vs1.42 ± 0.4 L, *p* = 0.008; FEV1: 1.08 ± 0.2vs1.25 ± 0.4 L, *p* = 0.045; FEF25-75: 1.31 ± 0.3vs1.43 ± 0.2 L/s, *p* = 0.009. NS: FVC: 1.23 ± 0.3vs1.31 ± 0.4 L, ns; FEV1: 1.09 ± 0.3vs1.25 ± 0.2 L, *p* = 0.001; FEF25-75: 1.35 ± 0.3vs1.51 ± 0.4 L/s, *p* = 0.027). Significant Rint reductions were also measured (HS: Rint exp.: 1.51 ± 0.2vs1.25 ± 0.2 KPa, *p* < 0.0001; Rint insp.: 1.40 ± 0.2vs1.26 ± 0.2 KPa, *p* = 0.018. NS: Rint exp.: 1.49 ± 0.4vs1.23 ± 0.2 KPa, *p* = 0.029; Rint insp.:

1.35 ± 0.2vs1.19 ± 0.2 KPa, *p* = 0.001).

After a 4-weeks treatment with HS, as compared to baseline values, only an improvement of %predicted FVC (*p* = 0.048) was found (Table 2). Worsening of %predicted FVC (*p* = 0.01), FEV_1_ (*p* < 0.0001), %predicted FEV_1_ (*p* < 0.0001), %predicted FEF_25-75_ (*p* = 0.001), FEF25-75 (*p* = 0.012) and expiratory Rint (*p* = 0.02) was detected in NS groups (Table 3).

**Table 2:** Lung function measured at time 0 (T0), after 4 (T4) and 16 weeks (T16) in the 12 patients who inhaled hypertonic saline

	T0	T4	T16	T0vsT4	T0vsT16
FVC (l)	1.42 ± 0.39	1.45 ± 0.39	1.49 ± 0.42	ns	*p* = 0.02
FVC % pred	89.2 ± 8.3	90.7 ± 7.7	91.9 ± 8.3	*p* = 0.048	*p* = 0.02
FEV_1_ (l)	1.25 ± 0.35	1.16 ± 0.23	1.25 ± 0.38	ns	ns
FEV_1_% pred	87.1 ± 9.5	85.7 ± 8.3	89.6 ± 9.6	ns	*p* = 0.09
FEF_25-75_ (l/s)	1.43 ± 0.22	1.42 ± 0.36	1.47 ± 0.39	ns	ns
FEF_25-75_% pred	77.0 ± 9.7	74.8 ± 5.6	77.1 ± 5.9	ns	ns
Rint exp. kPa(l/s)	1.25 ± 0.19	1.30 ± 0.22	1.24 ± 0.19	ns	ns
Rint insp. kPa(l/s)	1.26 ± 0.18	1.28 ± 0.16	1.21 ± 0.20	ns	ns

Data were expressed as mean values ± SD

**Table 3:** Lung function measured at time zero (T0), after 4 (T4) and 16 weeks (T16) in the 12 children who inhaled normal saline

	T0	T4	T16	T0vsT4	T0vsT16
FVC (l)	1.31 ± 0.36	1.23 ± 0.28	1.29 ± 0.35	ns	ns
FVC % pred.	89.7 ± 8.3	85.4 ± 9.3	87.9 ± 9.1	*p* = 0.01	ns
FEV_1_ (1)	1.25 ± 0.25	1.07 ± 0.20	1.14 ± 0.24	*p* < 0.0001	*p* < 0.0001
FEV_1_% pred	90.7 ± 8.6	85.1 ± 7.9	87.8 ± 6.7	*p* < 0.0001	ns
FEF_25-75_ (l/s)	1.51 ± 0.39	1.30 ± 0.24	1.40 ± 0.30	*p* = 0.012	ns
FEF_25-75_% pred	78.7 ± 6.1	65.9 ± 9.7	72.1 ± 9.4	*p* = 0.001	*p* = 0.019
Rint exp. kPa (l/s)	1.23 ± 0.17	1.50 ± 0.33	1.29 ± 0.19	*p* = 0.02	ns
Rint insp. kPa (l/s)	1.19 ± 0.19	1.37 ± 0.49	1.26 ± 0.21	ns	ns

Data were expressed as mean values ± SD

After a 16-weeks treatment with HS, as compared to baseline values, an improvement of FVC (*p* = 0.02) and %predicted FVC (*p* = 0.02) was found. A favorable trend of FEV1 was registered, although it failed to reach statistical significance. The mean values of expiratory and inspiratory resistance remained substantially unchanged (Table 2). On the contrary, a significant worsening of FEV_1_ (*p* < 0.0001) and FEF_25-75_ (*p* = 0.019) were found after the treatment with normal saline (Table 3).

The mean FVC, FEV1 and FEF25-75 improvements as well as expiratory and inspiratory Rint reductions, between T0 and T16 were higher in HS than in NS group, however, these were not-significant (data not shown).

**Fig. 2:**
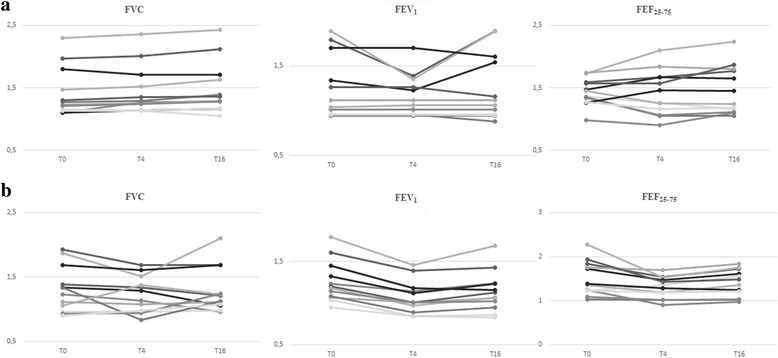
Individual changes in in FVC (L), FEV1 (L) and FEF25-75 (L/s) at the baseline (T0), at T4 and T16 for HS (**a**) and NS (**b**) groups of children

Figure 2 graphically represents the mean absolute difference in FVC, FEV1, FEF25-75 and expiratory and inspiratory Rint from the baseline at T0, T4 and T16 for the two treatment groups. Inspection of the data showed that spirometric and Rint parameters appeared to constantly improve at T0 and T16 in HS group but remained essentially unchanged at T4 and slightly improved at T16 in NS group.

No serious adverse events occurred in both groups. In the HS group, cough was observed (two patients) and, generally, disappeared after the first administration of HS, while one child complained about the unpleasant taste.

## Discussion

The present double-blind, randomized crossover study on 12 preschool children with cystic fibrosis, in clinically stable conditions, shows that the inhalation of HS twice daily is a safe treatment. To our knowledge, this is the first study testing Rint technique, an objective measurement of lung function in this range age [10, 12–14], over a course of HS treatment in children with cystic fibrosis. Our data, seems to confirm, on a little young population in the early phase of lung involvement, the short and middle-term benefits of HS, widely described in literature on adult patients [1, 2, 7, 15, 16]. In fact, a significant improvement of spirometric parameters was registered after 16-week HS therapy, while a worsening of those parameters was found in NS group. Similarly, Rosenfeld et al. have demonstrated a significant improvement of FEV0.5 in the group of 7% HS children younger than 6 years with cystic fibrosis, even if a reduction of pulmonary exacerbations was not reached [6]. These small changes may reflect a trend of improvement in airway performance that may result in clinically relevant implications. Subbarao et al. [4] was the pilot study that analyzed the utility, safety and tolerability of HS in children. The sole previous study on lung functions (by spirometry and the raised volume rapid thoracoabdominal compression technique) in preschool children (5.7 ± 0.8 years) with cystic fibrosis who had received HS therapy was by Dellon et al. [5].

The favorable effect of inhaled HS on airway is carried out by several postulated molecular mechanisms: HS breaks ionic bonds within the mucus and shields the negative charges thereby reducing the viscosity of secretions; it rehydrates airway surface improving mucus rheology; finally, it induces cough [1, 17, 18]. Moreover, HS contributes to the improvement of expiratory as well as inspiratory reduction of airway resistances by reducing the inflammatory edema of the airway.

In our small series, HS proved to be a safe and tolerable treatment. The most common adverse event expected with the introduction of the therapy were cough, throat irritation and unpleasant salty taste [2]. In our study two patients referred an increase of cough and one complained about oropharyngeal irritation. Cough typically decreased over time. However, we used a premedication with bronchodilator to prevent airway bronchoconstriction.

The present study may be of interest also from a methodological point of view, because it is the first to utilize the interrupter resistance technique, the gold standard for this age range, to assess lung function in preschool children. In fact, measurement of lung function in young children may be difficult and is prone to an increased failure rate and increased variability. Conventional lung function tests such as spirometry and body plethysmography have limited application because these techniques require a high degree of understanding and cooperation of the subject [19, 20].

Children in this age group are too old to be sedated but are unable to actively cooperate in many of the physiological maneuvers required for the lung function tests. Probably for this reason, statistically significant results of FEV1 with the spirometry have not been obtained.

Rint and spirometry reflect different aspects of the lung function and are not exactly interchangeable: Rint is more affected by large airway function and it measures the total resistance system and some added chest wall resistance. Moreover, it is a quick, noninvasive measure of respiratory resistance during tidal breathing that may be more easily performed in the preschooler than spirometry [21, 22]. This technique appeared as a reliable tool in the diagnostic work-up of pulmonary function in young CF patients [10, 12–14], even if its use that has been standardized by the ERS/ATS task force reported little evidence supporting the clinical utility in the preschool population with CF [22]. We consider that Rint and spirometry could play a role in the evaluation of pre-school children with CF because they may improve our ability to detect and treat early lung disease and exacerbations, particularly in pediatric respiratory centers where other more expensive techniques are not available.

Nowadays HS therapy may not be considered as an alternative treatment to rhDNase who has demonstrated a greater ability to improve lung function [3, 23–25] and to reduce pulmonary complications, but we should always keep in mind that rhDNase therapy is expensive, and its use is currently restricted in many countries only for patients with moderate or severe respiratory effort.

The treatment with hypertonic solution is quite cheap and safe and permitted at least a short and middle-term improvement of the respiratory function. The recent availability of the formulation of HS together with hyaluronic acid may be helpful in further avoiding the cough and the burning sensation consequent to the sole HS [26]. However, in children with CF, to avoid the potential risk posed by contamination of HS solutions we should recommend to use sterile unit-dose formulations [27].

Some limitations may be identified in our study, particularly the small number of the series, the single-center enrollment and a lack of wash-out period between the baseline measures.

Moreover, we decided to evaluate CF children in stable conditions with quite normal spirometric and Rint parameters at the enrollment. Finally, we limited the lung function tests to spirometry and Rint, insofar as multiple-breath inert gas and forced oscillation technique were not available.

## Conclusion

Seven percent hypertonic saline therapy seems to be a useful and safe treatment in young CF children with clinically stable conditions. Recently, the CF Foundation recommends that HS be offered to patients based on individual circumstances, either for chronic use or during acute pulmonary exacerbation [28]. Studies on larger series would be useful to define the effect of this therapy in particular on spirometric parameters and clinical outcomes.

## Abbreviations

CF: Cystic fibrosis
CFTR: CF transmembrane conductance regulator
HS: Hypertonic saline
NS: Normal saline
Rint: Interrupter resistance technique
